# Definition and application of indicators for urban bioeconomy: Analyzing the circularity maturity profile of european cities and regions

**DOI:** 10.12688/openreseurope.20022.1

**Published:** 2025-04-28

**Authors:** Marta Pereira-Ferrer, Alicia González-Míguez, Pedro Villanueva Rey, Teresa Alvariño Pereira, Miguel Ángel Suárez, Carla Bartolomé, David Silva, Jorge Rodrigues de Almeida

**Affiliations:** 1 CETaqua, Santiago de Compostela, Galicia, Spain; 2 Centro Tecnologico de la Energia y el Medio Ambiente, Cartagena, Region of Murcia, Spain; 3 Instituto Tecnologico del Embalaje Transporte y Logistica, Paterna, Valencian Community, Spain; 4 RdA Climate Solutions, Ovar, Portugal

**Keywords:** Circular Economy, Baseline Analysis, Urban Bioeconomy, Bioeconomy Indicators, Waste management, Urban Sustainability, Enabling Factors

## Abstract

**Background:**

The European Commission is promoting the Circular Economy (CE) to replace the linear model with one based on recycling and reuse. A genuine commitment to the CE requires that organizations and cities first identify and quantify the value leakages within their resource flows before designing and implementing any CE-related initiatives.

**Methods:**

This study presents an innovative set of Urban Bioeconomy Indicators (UBI) designed to assess and enhance CE initiatives in cities and regions, with a particular focus on biowaste management and valorization. Addressing the need for standardized yet locally adaptable metrics, this research proposes a comprehensive methodology for evaluating urban bioeconomy performance.

The study’s methodology involved an extensive review of existing CE indicators, followed by a structured selection and adaptation process. This led to the development of 20 indicators across six key dimensions: Waste, Water, Energy, Economy, Society, and Policy. The UBI set uniquely integrates both quantitative and qualitative indicators, including 'enabling factors' that capture crucial but hard-to-quantify aspects of CE implementation.

**Results:**

Key innovations of this research include: (1) a specific focus on urban bioeconomy, (2) emphasis on material valorization over energy recovery, (3) introduction of self-sufficiency indicators, and (4) incorporation of qualitative enabling factors. Nevertheless, the indicators are designed to measure both citizen behavior and waste management system performance, providing a holistic assessment of urban CE progress.

**Conclusions:**

By providing a standardized yet flexible methodology, this work contributes to the advancement of urban bioeconomy assessment. It serves as a valuable tool for local authorities to measure, compare, and enhance their bioeconomy performance, potentially laying the groundwork for future urban CE evaluations and policy development.

## Plain Language Summary

This study introduces a new set of Urban Bioeconomy Indicators (UBI) to help cities manage and improve how they handle biowaste (organic waste) in a circular economy (a system that reduces waste and reuses materials). The goal is to create standardized but flexible measures that work in different urban areas. To develop these indicators, researchers reviewed existing ones, selected the most relevant, and adapted them. The final set includes 20 indicators in six categories: Waste, Water, Energy, Economy, Society, and Policy. These indicators mix numbers (quantitative data) and descriptive aspects (qualitative data) to capture hard-to-measure factors that influence circular economy efforts. Key contributions of this study include: A focus on the urban bioeconomy (how cities handle organic resources). Prioritizing material reuse over just generating energy from waste. Creating self-sufficiency indicators (measuring how well cities manage resources independently). Including qualitative factors to understand citizen behavior and government policies. This set of indicators helps cities track their progress, compare results, and improve waste management and sustainability policies. It provides a useful tool for decision-makers to support better environmental strategies in urban areas.

## Introduction

Over the past few years, the European Commission has been implementing a broad array of policy initiatives related to the Circular Economy. These measures aim to facilitate the transition from a traditional linear "take-make-consume and discard" economic model towards a more sustainable "recycle-and-reuse" approach.

The goal is to mitigate the detrimental impact of economic activities on the environment and natural resources. Genuine engagement with the Circular Economy (CE) necessitates that organizations and cities first identify and quantify the value leakages occurring in their resource flows before designing and implementing any CE-related initiatives.

This value loss assessment requires an understanding of the current circularity level and the points at which these leakages occur, enabling the measurement of the gap between the existing scenario and the desired circular state.

The CE is an umbrella concept that embraces different approaches aiming to extend the value and utility of products and that consider waste as a valuable secondary resource. Although intuitive, the complexity of the CE concept produces an evolving debate on definitions, limitations, and boundaries ^1^. This fact has major implications for defining the most appropriate method for the assessment of the current circularity maturity profile of a given organization or city.

In this context, this research established a comprehensive assessment method to analyze city- and region-wide progress towards the CE. To this end, a set of CE indicators will be defined and applied to assess the current level of circularity in eight cities that foster this model through urban biowaste valorization. These indicators will serve as a benchmark for measuring circularity at the European regional level. Developed within the framework of the European Project HOOP [H2020 ID 101000836], these indicators support its goal of unlocking urban circular bioeconomy initiatives and fostering local bio-economies in eight European cities and regions. The project focuses on valorizing the organic fraction of municipal solid waste and urban wastewater sludge, transforming them into high-value products beyond compost and biogas

To accurately assess whether a specific intervention is achieving the desired change, it is essential to establish a baseline scenario. This allows for impact analysis and comparison with future scenarios, incorporating the application of the indicators to evaluate progress over time. Taking this into account, a baseline analysis could be useful for planning, monitoring and evaluating the circular measures.

**Figure 1.**
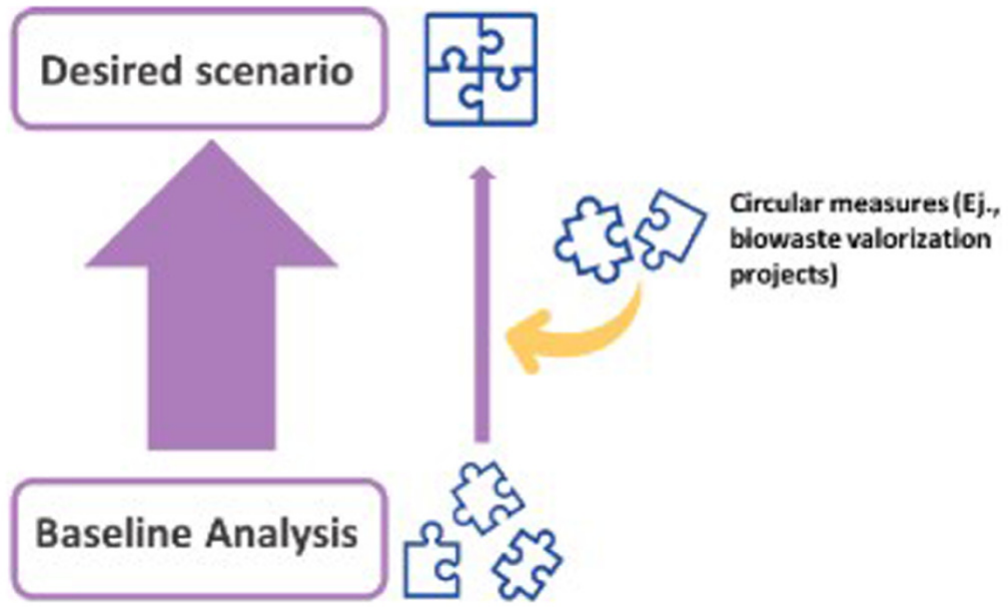
Baseline analysis philosophy.

According to this, the success of CE will be, to a large extent, measured in relation with the defined baseline. Therefore, a careful and correct baseline specification is crucial to assure an accurate impact estimation ( Figure 1). Among all the parameters to be considered, specific variables (e.g., circularity aspect), sources (e.g., industry, public entities, general data bases), geographic resolution, and years covered should be highlighted ^2^. Thus, depending on the problem to be dealt with, different thematic pattern scenarios could be built according to the individual issue or topic covered (issue-based scenarios) as well as classified according to the selected viewpoint (macro-, meso- and microlevels) ^3^.

The baseline analysis offers a macro-level picture (city/region level) picture about the urban circular bioeconomy maturity profile of the 8 different cities.

Having a solid system of indicators for assessing the CE implementation is key in order to achieve sustainable development. At present, there is a lack of a consensual methodology for the evaluation of the CE performance and progress, there is not a harmonized framework, which leads to an availability of a wide variety of indicators that could compromise the robustness of the analysis ^4, 5^.

According to the Organisation for Economic Co-operation and Development (OECD) report entitled “Inventory on Circular Economy Indicators” ^6^, CE indicators could be classified in the following categories: Economy and Business, Environment, Governance, Infrastructure and Technology and Jobs. In said report, the OECD reveals the following: environmental-related indicators prevail, especially energy usage or emissions; solid waste sector prevails over other industrial segments; lack of governance-related indicators; few city-level indicators available. Although the waste axis is overrepresented, most of the indicators in that sector are not defined from a closing-the-loop point of view; there is a lack of monitoring the reduction progress, which is the first approach of the 3R rule ^7, 8^.

The baseline analysis provides an overview of the current status of the 8 cities and regions previously mentioned, where circular economy measures are being implemented, allowing for the selection of the most appropriate improvements for each of them. The baseline analysis has been run in two levels:

- Baseline studies. Description of each city/region in different dimensions, presenting the main characteristics and the picture based on the six dimensions (Economy, Energy, Policy, Society, Waste, Water).
- Urban Bioeconomy Indicators. A set of indicators have been defined and selected in order to establish the reference point for each city/region.

## Methods

The methodology employed in this study followed a pragmatic, criteria-driven selection and adaptation process, focusing on tailoring existing circular economy (CE) indicators to urban contexts within a bioeconomy framework. The development of the indicators was grounded in the analysis of different cities ( Table 1) and their circularity-related activities. This allowed for the identification of key quantitative and qualitative indicators, as well as the creation of additional indicators addressing underrepresented categories, particularly in policy, society, and economy.

**Table 1.** Description of the Cities and Region Analyzed to Determine UBI.

*City*	*Population (hab)*	*Area (km ^2^)*	*Population density (hab/ km ^2^)*	*GDP per capita (€)*	*Predominant sector*	*Tourist/ hab*	*Geography*
1	~210,000	~250	~1,600	~40,000	Trade, logistics, financial services and tourism.	3.3	Coastal, near a major European capital with a modern urban planning.
2	~964,000	~2,040	~535	~35,000	Transforming industries, services, construction and tourism.	1.8	Coastal city in Southern Europe, built on hills along a river, with steep streets and ocean views.
3	~365,000	~138	~2,400	~70,000	Non-renewable energy production and ocean technology.	4.1	Coastal city in nordic country, surrounded by fjords and mountains.
4	~460,000	~880	~520	~27,000	Agriculture, tourism	1.2	Inland city in a Mediterranean climate.
5	~120,000	~1,700	~72	~50,000	Social and private forestry, and education.	1.2	Lakeside city in a northern European country, surrounded by forest.
6	~42,000	~59	~710	~38,000	Tertiary sector and industry (metallurgical, mechanical and electronic).	1.2	Inland city part of a metropolitan region with a mix of industry and nature.
7	~280,000	~9,500	~30	~18,000	Agriculture, forestry and phishing.	3	Mountainous region in southeastern Europe, with deep valleys, lakes, and highland plateaus
8	~315,000	~303	~1,050	~55,000	Services (insurance, banks, courts), ICT and universities.	2.1	Mid-sized inland city in the centre of Europe with 5 universities.

### Review of existing frameworks and data collection

The first stage involved a thorough review of prominent CE indicator sources, primarily the OECD's "Inventory on Circular Economy Indicators" and the "Indicators for circular economy transition in cities" report from the Urban Agenda for the EU ^4^. These sources provided a comprehensive starting point for identifying relevant indicators across various CE dimensions.

Beyond the existing data available from prior reports, additional information was necessary to complete the assessment of each city. To gather this knowledge, a structured technical questionnaire was distributed to city representatives. This questionnaire included key circularity aspects and was structured along six primary dimensions: Waste, Water, Energy, Society, Economy, and Policy. The questions were categorized into two levels—Primary and Secondary Questions—based on their relevance in establishing a robust baseline.

The key aspects covered in the technical questionnaire vary across the six dimensions previously mentioned. In the Waste sector, the required data highlights the proportion of organic fraction in municipal solid waste (OFMSW) and its diversion to landfills, along with the effectiveness of separate biowaste collection. It also tracks urban wastewater sludge (UWWS) generation, disposal methods, and agricultural reuse rates. Additionally, it includes metrics on municipal waste per capita, recycling rates, and biowaste management efficiency.

For the Water dimension, the focus extends to wastewater production, treatment, and reuse, encompassing household, industrial, and agricultural consumption. The wastewater treatment plant (WWTP) efficiency, the quality of discharged water, the volume of water withdrawn for potabilization, and the proportion of treated water returned to the environment are also assessed. The role of water management companies is also outlined.

In the Energy sector, the focus is on total energy consumption, renewable energy production, and energy recovery from biowaste and sludge. Additionally, it monitors fossil fuel dependence, greenhouse gas (GHG) emissions from waste transport and treatment, and the self-energy consumption of waste and water treatment plants. The survey also records impurities in selectively collected organic waste, which impact energy recovery efficiency.

The Society sector covers demographic and socioeconomic factors such as population density, unemployment rates, and main economic activities. It also tracks employment in waste management, environmental awareness initiatives, and eco-label certifications. This is because these factors influence waste generation, management efficiency and recycling participation.

The Economy sector highlights investments in circular economy projects and Research & Development (R&D) related to biowaste management and sludge treatment. Furthermore, it assesses the economic viability of waste recovery efforts, including job creation in the circular economy.

Finally, the Policy dimension evaluates the implementation of sustainability-related policies, waste treatment fees, and regulatory efforts aimed at enhancing circularity and resource recovery.

Data was collected from both public and private sectors to account for the complex and heterogeneous systems in which cities operate. One of the main challenges encountered during this process was the varying availability and accessibility of data across cities.

### Pre-selection and adaptation

The second stage involved pre-selecting indicators from the reviewed sources and adapting them to the urban bioeconomy context of the participating cities and regions. The goal was to ensure that the selected indicators captured the specific characteristics of urban circular bioeconomy, particularly in relation to biowaste valorization and bio-based resource flows. The indicators were distributed along the six identified dimensions (Waste, Water, Energy, Society, Economy, and Policy) to ensure comprehensive coverage.

### Optimization based on practical aspects

In the third stage, the pre-selected indicators were further refined based on practical implementation criteria. The key factors considered included:

- Data availability: Indicators were evaluated based on the accessibility and reliability of the required data sources within the participating cities and regions.
- Simplicity: Preference was given to indicators that were straightforward to calculate, use, and understand, promoting ease of implementation and interpretation.
- Comparability: Indicators were designed to allow for meaningful comparisons across diverse city types, considering differences in size, population, and socio-economic contexts.

This refinement process involved selecting and refining conventional indicators while also identifying categories or aspects that were underrepresented in existing frameworks. For example, as it was mentioned previously, the OECD’s report highlights the need for measurement of municipal circular economy initiatives or the need to assess energy recovery ^6^. As a result, new indicators were specifically developed to address these underrepresented dimensions. Consequently, the set of Urban Bieconomy Indicators was tailored to align with the project’s objectives and assumptions. However, this methodology provides a structured approach that can be integrated into other cities interested in adopting HOOP’s framework. These additional indicators were designed to enhance applicability and comprehensibility, ensuring that cities with diverse characteristics can effectively implement and benefit from them.

### Final criterion application

Finally, at this stage, a set of criteria was applied, considering the complexity of the urban circular bioeconomy. Some of these factors included:

- **Bio-based approach**: Indicators were designed to focus on biowaste and biological resources, aligning with emphasis on the urban circular bioeconomy.
- **Urban scope**: Indicators were tailored to capture the unique dynamics and challenges of urban environments, considering factors such as waste management systems, stakeholder involvement, and policy frameworks.
- **Circularity principles**: Indicators were selected and adapted to prioritize material valorization, waste prevention, and the recirculation of resources within the urban ecosystem.
- **Comparability**: Since the cities and regions have different sizes and number of inhabitants, several indicators have been corrected with the number of inhabitants enabling the comparison.
- **Enabling factors**: Qualitative indicators were incorporated to evaluate enabling conditions such as awareness campaigns, entrepreneurial initiatives, and policy support, which facilitate the transition to a circular bioeconomy.
- **Target of evaluation**: the indicators should allow for assessing the performance and commitment of both the management system and citizenship, as the circular urban bioeconomy depends on both.

The final set of UBI has been implemented into a digital tool available on the HOOP project’s web platform. This tool allows users to assess their city's circularity performance by inputting relevant data and generating a customized circularity score. By providing an accessible, open-access, user-friendly interface, this initiative aims to promote broader engagement and facilitate the practical application of the developed indicators. This tool is available at: HOOP Circularity Label.

By building upon existing CE indicator frameworks, analyzing them in relation to the specific needs of the study, and incorporating practical considerations and guiding principles, a tailored set of indicators was developed. These indicators serve as a benchmark for measuring initial status, establishing baselines, monitoring progress, and identifying areas for improvement in cities and regions aiming to implement urban circular bioeconomy initiatives. This analysis establishes the starting point for each city or region to enhance its bioeconomy performance, offering a structured methodology that can be adapted and expanded within broader circular economy transitions.

## Results and discussion

Even though CE is not clearly defined, as it is an umbrella concept, there is a need for specific methods to measure its progress, but it has to be done carefully since indicators could lead to incoherent conclusions. It is important that indicators represent two defining characteristics of CE, which differentiate it from linear economy: **slowing the use of resource** loops (e.g., extending product lifespans) and **closing resource loops** (e.g., ensuring material recovery and reuse) ^9^.

Previous studies have demonstrated that not one, but a set of indicators are necessary to evaluate CE ^9^, so the final 20 UBI are presented in Table 2, which were selected to assess key aspects of CE at the urban level. A distinctive feature of this framework is the inclusion of economic, policy, and social indicators, addressing dimensions that are often underrepresented in CE assessments.

**Table 2.** HOOP Urban Bioeconomy indicators.

*nº*	*Indicator*	*Area*	*Unit*	*Explanation*
1	Municipal biowaste production	Waste	kg/cap*year	Total amount of biowaste produced per capita (Post-consumer not included). Municipal biowaste production= (organic fraction from MMSW (mixed municipal solid waste) + separately collected urban biowaste + park and gardens waste)/ inhabitants
2	Percentage of biowaste separately collected	Waste	%	Biowaste collected separately with respect to total amount of biowaste (Post-consumer not included). % OFMSW separately collected = [(separately collected biowaste + park and gardens waste)/(organic fraction from MMSW + separately collected biowaste + park and gardens waste)] *100
3	Quality of biowaste streams	Waste	%	OFMSW quality= [100- (percentage of impurities on OFMSW collected separately)]
4	Rate of UWWS re-used	Waste	%	% of UWWS reused
5	Percentage of biowaste used for material recovery	Waste	%	% Total amount of biowaste intended to waste-to-material approach (Excluding incineration and impurities). % biowaste used for material recovery = (Total biowaste valorized / Total biowaste produced)*100
6	nº bioproducts produced from waste	Waste	nº	Quantity of bioproducts (biofertilizers, compost, etc.) produced from biowaste.
7	Biowaste management self-sufficiency	Waste	%	% of biowaste treated inside the City. Indirect indicator of infrastructure availability.
8	Implementation rate of biowaste separate collection system	Waste	% or qualitative	% of the city population with regular biowaste fraction collection (residential).
9	Water reuse	Water	%	% of water from wastewater treatment plants which is reused.
10	Wastewater production	Water	m3/cap*year	Wastewater production= Amount of wastewater generated / inhabitants.
11	Biogas production	Energy	m3/cap*year	Biogas production from urban biowaste, UWWS or wastewater treatment per inhabitant.
12	Costs associated with OFMSW and UWWS management	Economy	€/ton	Costs associated with urban biowaste and urban sewage sludge treatment.
13	Number of R&D projects related to biowaste management and treatment	Economy	nº	Excluding HOOP.
14	Financial mechanisms to reduce biowaste	Economy	Qualitative (yes/no)	Financial mechanisms aimed at reducing the generation of biowaste.
15	Promotion of circular economy entrepreneurial initiatives	Economy	Qualitative (yes/no)	City’s support to entrepreneurial initiatives in the circular economy.
16	Awareness rising campaigns about waste	Social	Qualitative (yes/no)	The city/region carries awareness campaigns about biowaste.
17	Coordination entity to engage stakeholders involved in CE	Social	Qualitative (yes/no)	The city/region counts on an entity/department that coordinates and engages stakeholders involved in circular economy.
18	Compost applicability in local agriculture/gardens	Social	Qualitative (yes/no)	The produced compost finds applicability in local agriculture/gardens.
19	Circular economy strategies in the city	Policy	Qualitative (yes/no)	Considering only those promoted at city level (EU, national excluded).
20	Innovation public procurement	Policy	Qualitative (yes/no)	The city/region has been involved in projects undertaken using innovative public procurement, namely PCP (pre-commercial procurement) or PPI (public procurement of innovative solutions).

### Key findings by dimension

The selection of indicators provides insights into both citizen behaviour and municipal waste management systems. For example, the selective collection rate and its quality serve as indicators of social awareness, while the implementation rate of selective collection systems evaluates the efficiency of municipal infrastructure. One of the key findings from this study is the high heterogeneity among cities and regions regarding circular economy performance. Differences in socio-economic conditions, regulatory frameworks, and infrastructure availability make it difficult to apply a one-size-fits-all approach. Some cities include private-sector waste in their reports, while others focus solely on municipal waste, further complicating direct comparisons.

**Figure 2.**
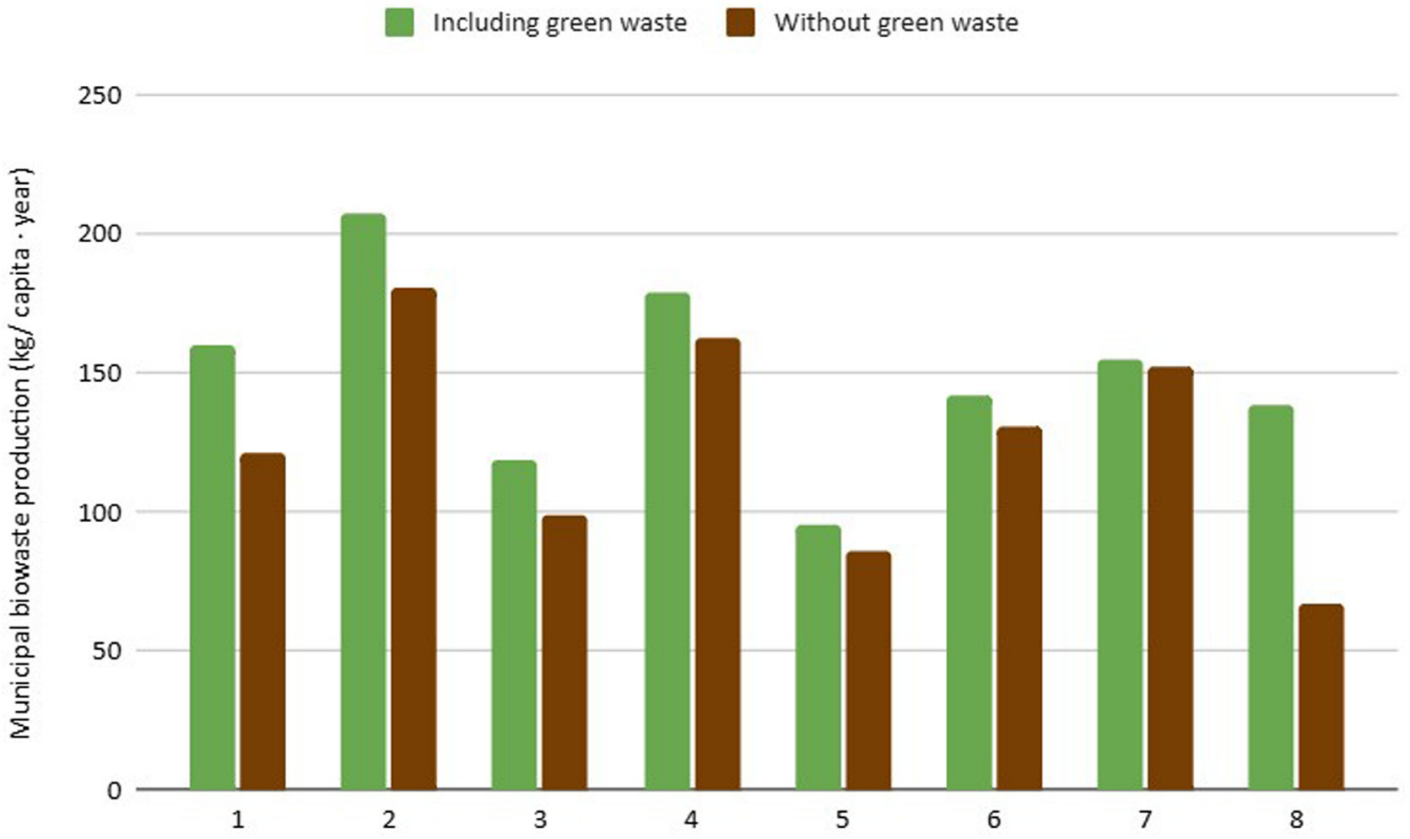
Urban biowaste production, including and excluding generated green waste.

**Figure 3.**
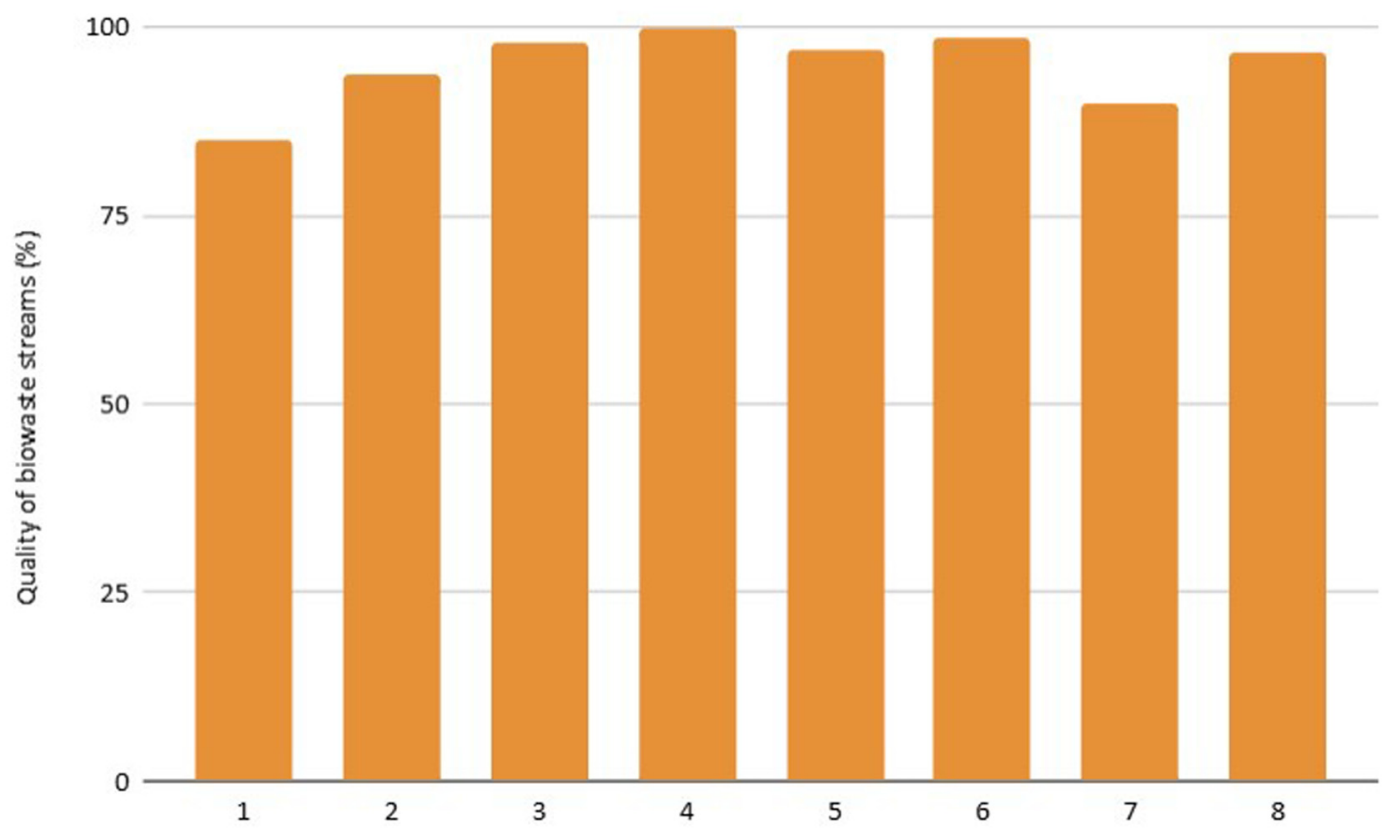
Quality of biowaste streams.

**Figure 4.**
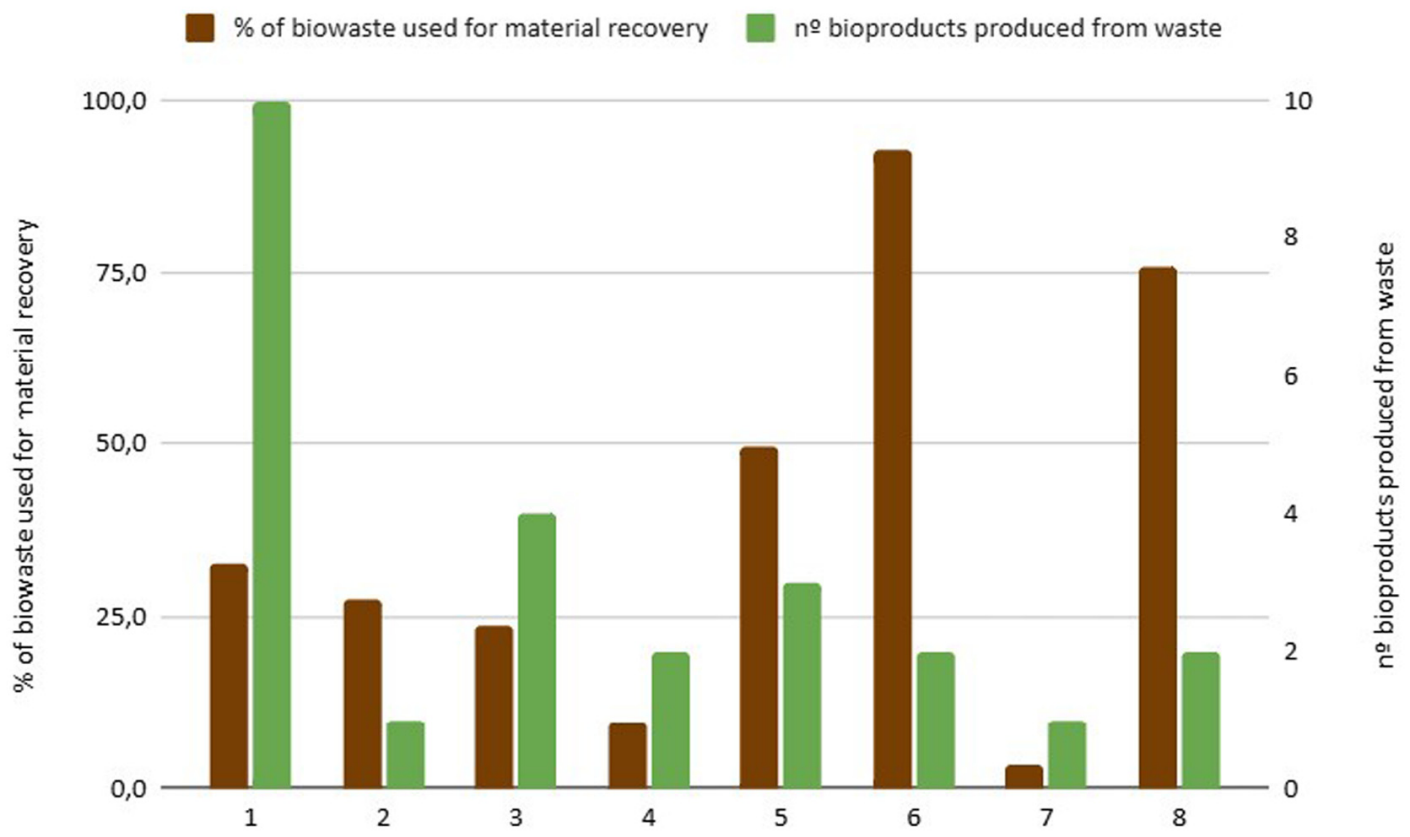
Percentage of biowaste used for material recovery and number of bioproducts generated from urban biowaste.

**Figure 5.**
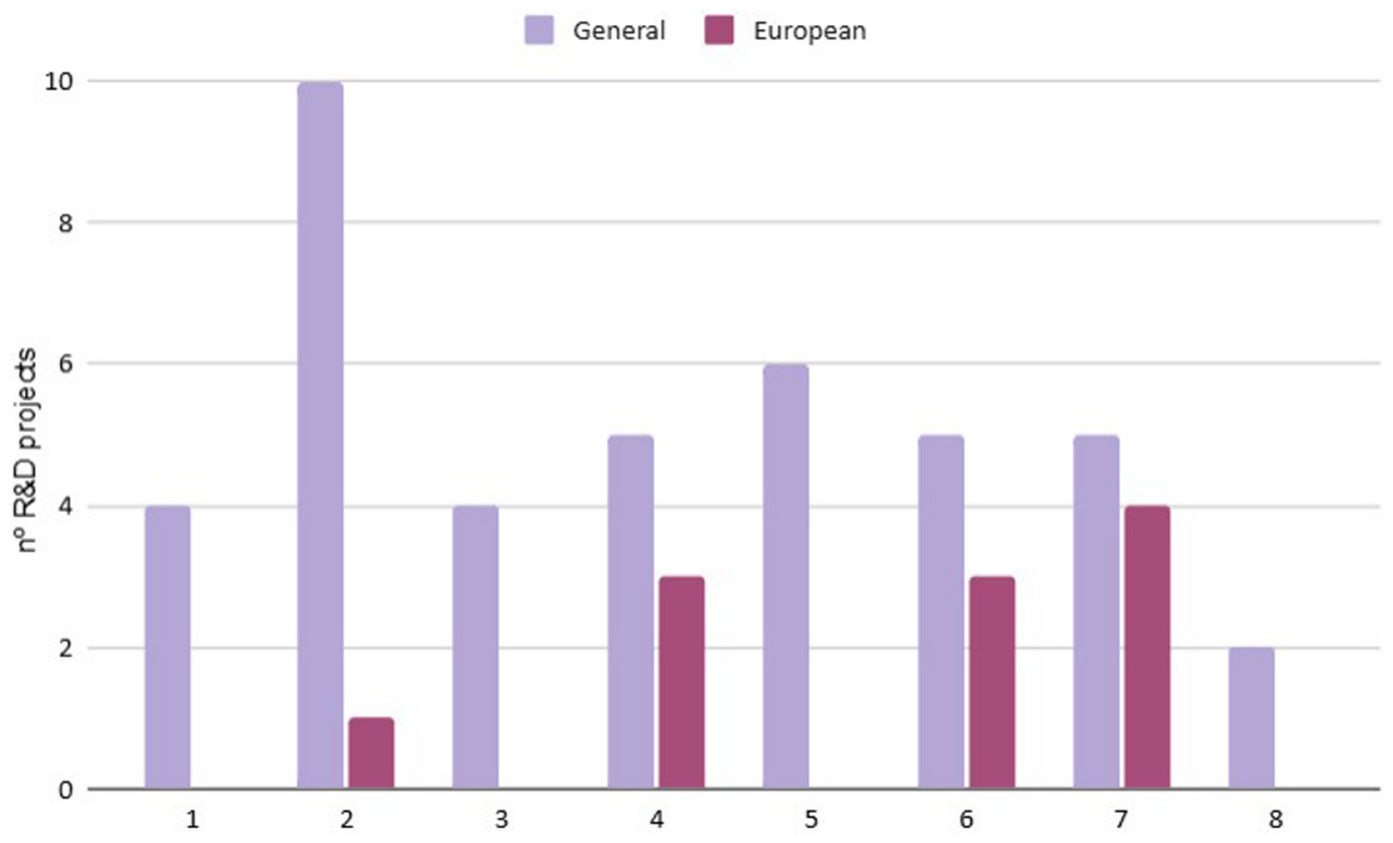
Number of R&D projects.

**Waste Dimension**: Waste-related indicators are the most represented due to their fundamental role in urban bioeconomy. It was observed that cities with extensive green areas generate higher amounts of biowaste, which influences the results ( Figure 2). Additionally, small changes in the composition of municipal waste can significantly alter the estimation of biowaste generation ( Figure 3). The selective collection rate and quality of the separated material depend on public awareness and municipal infrastructure, showing strong variability between cities. Furthermore, the biowaste selective collection and recovery indicator excludes organic waste fractions that go to home composting, as from 2027 onwards, this fraction will no longer be considered recycling. This is the case of city 5, which presents the lowest score in this indicator (96 kg/cap*year), as home composting is implemented in 48% of households.
**Material Valorization**: The percentage of biowaste used for material recovery differs depending on the available local infrastructure ( Figure 4). Notably, only City 7 reported that compost does not find applicability in local agriculture or gardens, which may indicate barriers in compost utilization or regulatory constraints specific to that region. In cities with advanced anaerobic digestion or composting technologies, material valorization reaches 100%. In contrast, those relying on incineration report nearly 0% material valorization, highlighting a gap in waste recovery capacity.
**Water Dimension**: Water-related indicators are less represented due to the difficulty in defining clear boundaries between water and waste management. This disparity makes data collection difficult, often requiring extrapolations and assumptions. For example, the reuse of water varies drastically among the evaluated cities, with some reaching reuses rates of 11% (city 4), while others do report reduced water reutilization (0.41% and 0.16% for cities 2 and 3 respectively) or no reutilization at all (rest of the cities). This disparity is due to differences in wastewater treatment infrastructure and the implementation of local regulations. Furthermore, the percentage of reuse is also related to the water availability in the region. The city number 4 is an inland city with Mediterranean climate that suffers from water scarcity, explaining the highest reuse rate.
**Energy Dimension**: Biogas production is a key indicator within this dimension, but the data show significant differences among cities. Only four of the eight cities (3–5 and 8) generated biogas, producing 7.34, 6.48, 33.16 and 17.56 m3/capita · year respectively. This suggests that the energy conversion capacity of waste is highly dependent on local investments in bioenergy technologies.
**Economic Dimension**: Investments in R&D show significant disparities between cities ( Figure 5). While some cities have implemented financial mechanisms to reduce biowaste generation and promote innovation, others lack effective economic incentives ( Table 3). Differences in support for circular economy entrepreneurship also highlight inequalities in access to funding and policy support. Moreover, the lack of standardization between technical and social projects complicates direct comparisons. By correlating the information provided in Figure 5 and Table 3, it becomes evident that the cities that do not promote financial or business mechanisms for economic circularity (cities 2, 4, 6 and 7) are those receiving the highest levels of European funding. Notably, these cities also have the lowest GDP per capita among the seven cities.
While some cities have implemented financial mechanisms to reduce biowaste generation and promote innovation, others lack effective economic incentives ( Table 3). Differences in support for circular economy entrepreneurship also highlight inequalities in access to funding and policy support. Moreover, the lack of standardization between technical and social projects complicates direct comparisons. By correlating the information provided in Figure 5 and Table 3, it becomes evident that the cities that promote financial or business mechanisms for economic circularity (cities 1, 3, 5 and 8) are those involved in more projects and activities of different nature than European projects. Notably, these cities also have the highest GDP per capita among the seven cities.
**Social and Policy Dimensions**: Qualitative indicators reveal that all cities conduct awareness campaigns on biowaste ( Table 4), but only half have coordinating entities to engage key stakeholders in the circular economy. This highlights the importance of public engagement in CE transition as the lack of well-defined political strategies limits the effective implementation of these programs. However, cities exhibit significant differences in their policy frameworks, making cross-regional comparisons challenging.

**Table 3.** Financial mechanisms and investment in R&D projects related to the circular economy.

		Cities/ regions							
Category	Indicator	1	2	3	4	5	6	7	8
Economic	Financial mechanisms to reduce biowaste	no	no	yes	no	yes	yes	yes	yes
Economic	Promotion of circular economy entrepreneurial initiatives	yes	yes	yes	yes	yes	no	no	yes

**Table 4.** Key Indicators for Circular Economy Engagement and Strategies in the Cities.

		Cities/ regions							
Category	Indicator	1	2	3	4	5	6	7	8
Social	Awareness rising campaigns about biowaste (y/n)	yes	yes	yes	yes	yes	yes	yes	yes
Social	Coordination entity to engage stakeholders involved in CE (y/n)	yes	yes	no	no	no	yes	yes	yes
Policy	Circular economy strategies in the city	yes	yes	no	no	yes	yes	yes	yes

## Conclusions

This study has developed an innovative set of Urban Bioeconomy Indicators (UBI) to assess cities’ performance in the circular economy, with a particular focus on biowaste management. By integrating both quantitative and qualitative indicators, this framework enables a comprehensive evaluation across six key dimensions: Waste, Water, Energy, Economy, Society and Policy. The resulting set of 20 indicators, including seven enabling factors, provides a clear perspective on the practices that facilitate a city or region’s transition to a CE. These enabling factors are particularly important, as they represent best practices that support the shift toward a more sustainable, bioeconomy-driven circular model. Ultimately, the goal is to empower informed decision-making, allowing cities to implement targeted strategies to enhance their circular bioeconomy performance.

The UBI framework represents a more specialized, locally-focused, and bioeconomy-oriented approach compared to the general reports from the OECD and the Urban Agenda for the EU. Unlike these broader frameworks, the UBI is specifically tailored to address urban bioeconomy and biowaste management. By providing a standardized set of measurements, the UBI offers a tool for tracking a city’s progress over time and enables comparisons across different cities or regions. This tailored approach makes it easier to assess the development of a city’s bio-based circular economy.

The indicators developed address a critical gap identified in the OECD report, which lacked indicators specifically applicable at the city or regional level. Consequently, the UBI provides local authorities with a valuable tool to measure and enhance their bioeconomy performance. This study’s framework could serve as the foundation for a more standardized methodology, an area that has been underdeveloped in current research ^4, 5^.

The proposed UBI structure categorizes indicators into six dimensions (above mentioned), diverging from the OECD's five-category framework ^6^ of Economy and Business, Environment, Governance, Infrastructure and Technology, and Employment. This distinction reflects the specific focus on the urban bioeconomy, with the added benefit that the “suggested indicators are only meant to support discussions and further work on CE indicators at the city level” ^7^. This more focused approach is essential for capturing the nuances of urban bioeconomy systems and their potential for sustainable development.

Moreover, the UBI framework presents additional improvements over existing indicators, offering a more refined and context-specific approach to assessing urban circular economy performance.

1. **Inclusion of qualitative "enabling factors"**: UBI incorporates qualitative indicators in the social and political dimensions, capturing critical elements like public engagement, political commitment, and social acceptance, which are often overlooked in traditional quantitative models.
2. **Focus on material valorization of biowaste**: UBI emphasizes material recovery over energy recovery, aligning with circular economy principles. Indicators such as the "Percentage of biowaste used for material recovery" and "Number of bioproducts produced from waste" promote a more sustainable and resource-efficient approach.
3. **Specific biowaste management indicators**: UBI includes precise indicators on biowaste management, such as the quality and implementation rate of separate collection, offering more detailed insights into waste management effectiveness than general indicators.
4. **Introduction of self-sufficiency indicators**: UBI introduces self-sufficiency indicators to measure a city’s capacity to manage its own biowaste, providing a fresh perspective on local waste management that traditional indicators often miss.
5. **Enhanced evaluation of project impacts**: The UBI framework serves as a powerful tool for evaluating the progress and impact of specific circular economy projects implemented in participating cities and regions, allowing for a clearer understanding of the effectiveness of circular economy initiatives and offering a more detailed approach than generalized circular economy indicators that fail to capture local dynamics and project-specific results.
6. **Specialized, locally-relevant approach**: UBI is tailored to address the unique needs of urban bioeconomies, supporting more effective policies, targeted interventions, and the transition to sustainable, bioeconomy-driven urban systems, in contrast to broader, one-size-fits-all models.
7. **Different levels of progression towards CE**: the baseline analysis, applying the set of UBI, shows that cities are at different levels of development across various analyzed dimensions. For example, some cities have been selectively collecting biowaste for decades, while others are just beginning. Furthermore, every city excels in at least one specific dimension.

## Data Availability

No data are associated with this article.
