# Supplementary material for: Definition and application of indicators for urban bioeconomy: Analyzing the circularity maturity profile of european cities and regions
Source: Open Res Eur. 2026 Feb 10;5:116. Originally published 2025 Apr 28. [Version 2] doi: 10.12688/openreseurope.20022.2 (PMC12946700; doi:10.12688/openreseurope.20022.2)
Supplement: Peer review history

# Reviewer report: Reviewer response for version 2

**DOI:** 10.21956/openreseurope.24928.r69545

**Published:** 2026-03-02

**Authors:** Nurliyana Abdul Raof

**Affiliations:** 1 Universiti Teknologi PETRONAS, Perak, Malaysia

Full Report

 Overall, this is a relevant and timely methods paper. The focus on the urban bioeconomy, especially biowaste and wastewater valorisation, is well aligned with current circular economy (CE) priorities at European level. I appreciate the effort to move beyond purely environmental indicators and include enabling factors (policy, social and economic dimensions), which are often missing in CE assessments.

 However, since this is presented as a methods article, the main expectation is transparency and reproducibility. At the moment, the paper is strong in concept and indicator design, but some important methodological and technical details are still missing.

**Technical soundness**

 The overall approach (review of existing frameworks → pre-selection → adaptation → refinement based on practical criteria → final set of 20 indicators) is logical and pragmatic. Table 2 is useful, and several quantitative indicators include formulas. **However, for a methods paper, some technical aspects need to be more clearly specified:**

- How exactly were indicators selected from the OECD and Urban Agenda frameworks?
- Was there an expert validation process or structured selection criteria?
- How were inconsistencies across cities (municipal vs private waste, home composting, green waste inclusion) harmonised?

**Most importantly, the paper mentions a “circularity score” and a maturity profile, but it does not clearly explain:**

- How indicators are normalised
- Whether they are weighted
- How qualitative indicators are combined with quantitative ones
- How the final score or label is calculated

This part is essential and needs to be explicitly described.

**Replication**

 Indicator definitions are provided, which is good. However, the method is not yet fully replicable.

 To improve this, I suggest:

- Including the **technical questionnaire** as **supplementary material**
- Providing a clear step-by-step explanation of the calculation process
- Adding a simple worked example (for one city) showing raw data

Without this, it is difficult for other researchers or cities to apply the method independently.

**Data availability**

 The manuscript presents results, but the underlying dataset is not provided. Even if some raw data are confidential, the authors should at least provide:

- The final city-level indicator values used in the figures/tables
- Reference year (s)
- General data sources and any assumptions used

If confidentiality applies, this should be clearly stated, and aggregated or processed values should still be shared to allow reproduction of the published figures.

**Digital tool clarification (Important)**

 The manuscript states that the final set of UBI has been implemented in a digital tool available on the HOOP project’s web platform, but the link provided cannot currently be accessed.

 This is important, since the tool seems to play a key role in generating the circularity score.

 I recommend that the authors:

- Briefly describe what type of tool has been used (e.g., web-based dashboard, Excel-based calculator, custom-built software, open-source platform, etc.)
- Explain how the scoring logic is implemented within the tool
- Clarify whether the tool is publicly accessible, and if not, provide at least a methodological description of its backend logic

Without this clarification, it is difficult to evaluate the robustness and transparency of the scoring system.

**Major points to address**

1. Clearly explain how the circularity score / maturity profile is calculated (normalisation, weighting, aggregation).
2. Provide the technical questionnaire and calculation procedure as supplementary material.
3. Share at least the processed city-level dataset used in the results.
4. Clarify the digital tool (type, accessibility, and scoring logic), especially since the provided link cannot currently be viewed.
5. Define boundary and harmonisation rules more clearly to improve comparability.

Is the rationale for developing the new method (or application) clearly explained?

Yes

Is the description of the method technically sound?

Partly

Are the conclusions about the method and its performance adequately supported by the findings presented in the article?

Partly

If any results are presented, are all the source data underlying the results available to ensure full reproducibility?

No

Are sufficient details provided to allow replication of the method development and its use by others?

Partly

Reviewer Expertise:

NA

I confirm that I have read this submission and believe that I have an appropriate level of expertise to confirm that it is of an acceptable scientific standard, however I have significant reservations, as outlined above.

# Reviewer report: Reviewer response for version 2

**DOI:** 10.21956/openreseurope.24928.r69372

**Published:** 2026-02-26

**Authors:** Jaime Solis-Guzman, Nadia Falah

**Affiliations:** 1 Department of Architectural Constructions II, University of Seville Higher Technical School of Engineering, Seville, Andalusia, Spain

The authors have worked diligently to improve the conceptual framework and better explain its limitations, which is a positive step. While we believe further elaboration on the indicator selection process, the formal description of the standardization procedures, and the analysis of non-descriptive results would be necessary, the article demonstrates a sufficiently significant improvement to be considered acceptable for indexing.

Is the rationale for developing the new method (or application) clearly explained?

Partly

Is the description of the method technically sound?

Partly

Are the conclusions about the method and its performance adequately supported by the findings presented in the article?

Partly

If any results are presented, are all the source data underlying the results available to ensure full reproducibility?

Partly

Are sufficient details provided to allow replication of the method development and its use by others?

Partly

Reviewer Expertise:

Circular cities, life cycle assesment

We confirm that we have read this submission and believe that we have an appropriate level of expertise to confirm that it is of an acceptable scientific standard.

# Reviewer report: Reviewer response for version 1

**DOI:** 10.21956/openreseurope.21657.r60188

**Published:** 2025-10-07

**Authors:** Gurudas Nulkar

**Affiliations:** 1 Gokhale Institute of Politics and Economics, Pune, Maharashtra, India

The article needs to carry more discussion on the indicators, how they were arrived at, and why are they useful. I don’t quite get the indicators and their utility in circular economy. The indicators are not new, and certainly not exhaustive. The authors need to write why they feel the indicators offer a better picture. Figure 1 does not convey any real meaning.

 I feel this article is good for academic usage, but not quite useful for practitioners, unless the authors can provide guidelines for practical usage.

Is the rationale for developing the new method (or application) clearly explained?

Partly

Is the description of the method technically sound?

Partly

Are the conclusions about the method and its performance adequately supported by the findings presented in the article?

No

If any results are presented, are all the source data underlying the results available to ensure full reproducibility?

No

Are sufficient details provided to allow replication of the method development and its use by others?

Yes

Reviewer Expertise:

Circular economy

I confirm that I have read this submission and believe that I have an appropriate level of expertise to confirm that it is of an acceptable scientific standard, however I have significant reservations, as outlined above.

# Response

**Published:** 2026-01-30

**Authors:** Teresa Alvariño Pereira

Thank you for your review and valuable feedback. Regarding the first point of your comments, the indicators presented in the paper were developed within the framework of the HOOP project, following a structured co-creation process. The selection and validation of the indicators were conducted through a technical questionnaire distributed among the eight Lighthouse Cities and their respective Local HOOP Committees. This process involved consultation with a range of local experts and stakeholders, including municipal authorities, public utilities, and private actors, to ensure that the indicators reflected both scientific and operational perspectives on urban circular bioeconomy performance.  The detailed results of this consultation are included in an internal confidential deliverable of the project and therefore cannot be publicly available. Nevertheless, the methodology followed standard expert elicitation practices and ensured that the resulting set of indicators was context-specific, comparable across urban settings, and aligned with the objectives of supporting circular bioeconomy implementation at the city level. Regarding your observation that indicators are not new or exhaustive, we fully agree. However, the intention of this work was not to create novel metrics per se, but to identify and adapt the most suitable and operational indicators for assessing circularity in cities with diverse socio-economic and environmental contexts. The selection was therefore based on their relevance, applicability and robustness for urban bioeconomy assessment rather than on conceptual novelty. In this sense, we consider the resulting set to be comprehensive and replicable across cities with similar characteristics. The framework integrates both quantitative and qualitative indicators covering six dimensions, allowing a holistic evaluation of circularity performance and enabling consistent benchmarking between urban contexts. We also agree with the comment that Figure 1 did not convey sufficient meaning. Accordingly,  it has been removed from the revised version. Finally, we appreciate your remark that the paper is primarily academic in nature. Nonetheless, the proposed framework is also applicable to practitioners, particularly for cities and regions with similar characteristics to those analyzed in the study. The set of indicators enables an initial evaluation of circularity performance, helping local authorities identify baseline conditions, detect priority areas for improvement, and monitor progress over time. In this sense, the indicator framework is not intended as a purely theoretical exercise, but as a practical decision-support tool that can be replicated and adapted by the municipalities aiming to implement or enhance circular bioeconomy strategies.

# Reviewer report: Reviewer response for version 1

**DOI:** 10.21956/openreseurope.21657.r60195

**Published:** 2025-10-07

**Authors:** Jaime Solis-Guzman, Nadia Falah

**Affiliations:** 1 Department of Architectural Constructions II, University of Seville Higher Technical School of Engineering, Seville, Andalusia, Spain

The manuscript addresses an important and timely topic, proposing a set of **Urban Bioeconomy Indicators (UBI)** to assess and enhance circular economy (CE) initiatives in cities and regions. The focus on urban bioeconomy and the attempt to combine quantitative and qualitative indicators is innovative and of potential value for both academic and policy audiences. The paper is aligned with the European Commission’s CE agenda and has clear relevance for local authorities and practitioners.

**There are some points that must be mentioned: **

1. The proposed UBI framework is well aligned with the existing gaps highlighted in OECD and Urban Agenda reports and represents a genuine innovation. Despite this alignment, the framework lacks a fully systematic methodology for indicator selection. Recent researches apply data-driven methods (machine learning, text analytics) for systematic indicator identification and classification. 
2. The integration of both quantitative and qualitative indicators—particularly the inclusion of “enabling factors”—is commendable as it captures social and institutional dimensions often overlooked in CE research. However, the selection and validation of qualitative factors are insufficiently explained, which limits the robustness and comparability of the results. More advanced analytical techniques could transform this process from a subjective exercise into a more rigorous, evidence-based methodology.
3. The development of a digital tool (HOOP Circularity Label) is a significant added value for policymakers and city stakeholders. Nevertheless, the technical functioning, underlying algorithms, and transferability of this tool to other cities are not clearly presented. 
4. The use of case studies across eight European cities moves the work beyond the theoretical level and increases its practical relevance. Yet, the analysis remains largely descriptive. Comparative and inferential analyses across cities are missing, which would have allowed stronger conclusions and more generalizable insights. 

**Some weaknesses and suggestions for improvement**

- **Transparency in indicator selection**: The paper states that indicators were chosen through review and adaptation but does not clarify the exact criteria for inclusion or exclusion. A flowchart or comparative table should be provided to demonstrate which indicators were considered, which were discarded, and on what basis. This would significantly improve the replicability of the study.
- **Data analysis**: The results section is primarily descriptive. More in-depth comparative or statistical analyses are needed to highlight differences and similarities across cities, as well as to identify which dimensions (waste, water, energy, policy, etc.) contribute most to performance variation.
- **Limitations**: Differences in data availability between cities, the uneven quality of collected data, and the challenges of comparing qualitative indicators are critical issues that need explicit discussion. Acknowledging these would add transparency and guide future research.
- **Policy relevance**: While the paper mentions policy implications, it lacks specific, actionable recommendations. A dedicated **Policy Recommendations** section would greatly increase the value of the study for non-academic audiences. For instance, it could highlight which categories of indicators are most relevant for local governments and how they can be operationalized in waste management or urban bioeconomy programs.
- **Definition of urban bioeconomy**: The manuscript introduces the term but does not provide a clear and distinct definition compared to the broader bioeconomy. Since the concept is still emerging, the literature review should systematically trace how previous studies have defined it, what gaps exist, and how this paper arrives at its own definition. 
- **Methodological weakness**: The study does not present an updated methodological structure for indicator identification, classification, normalization, and selection. The absence of such a systematic approach in this paper represents a methodological gap, and addressing it would significantly strengthen the contribution and scientific credibility of the work.

The manuscript makes a valuable contribution by highlighting the role of bioeconomy in urban circular economy strategies and by proposing a mixed set of indicators. However, the lack of greater methodological depth in the selection of indicators, insufficient analytical depth and the absence of clear policy recommendations reduce its impact. Substantial revisions—particularly in methodology and conceptual framing—are necessary to enhance the robustness, reproducibility, and policy relevance of the paper.

Is the rationale for developing the new method (or application) clearly explained?

Partly

Is the description of the method technically sound?

Partly

Are the conclusions about the method and its performance adequately supported by the findings presented in the article?

Partly

If any results are presented, are all the source data underlying the results available to ensure full reproducibility?

Partly

Are sufficient details provided to allow replication of the method development and its use by others?

Partly

Reviewer Expertise:

Circular cities, life cycle assesment

We confirm that we have read this submission and believe that we have an appropriate level of expertise to state that we do not consider it to be of an acceptable scientific standard, for reasons outlined above.

# Response

**Published:** 2026-01-30

**Authors:** Teresa Alvariño Pereira

We sincerely thank the reviewers for its insightful and thoughtful comments. We acknowledge that several aspects of the paper need clarification and we have addressed each of these points as follows:

- Transparency in indicator selection: As described in the Methods section, the indicators were created within the framework of the HOOP project through a structured co-creation process. Their selection and validation were conducted via technical questionnaires and expert consultations with representatives from the eight Lighthouse Cities and their respective Local HOOP Committees. This participatory process involved a wide range of stakeholders, ensuring that the indicators reflected both scientific knowledge and operational feasibility in different urban contexts. The detailed data and results from these consultations, including the specific inclusion and exclusion criteria applied to the initial list of indicators, are part of a confidential internal project deliverable and therefore cannot be publicly disclosed. Nevertheless, the process followed recognized expert elicitation practices, ensuring methodological rigor and comparability across cities. The final indicator set was thus validated through expert consensus, guaranteeing its relevance, consistency and replicability in similar urban environments.
- Data analysis: We acknowledge that the results presented are primarily descriptive, as the main objective of this study was to establish a baseline analysis for the participating cities and regions. The intention was to provide an initial, harmonized assessment of their circularity maturity profile rather than to perform an in-depth statistical or inferential analysis at this stage. The heterogeneity of the eight Lighthouse Cities, in terms of geographic context, population size, data availability and local governance structures, makes direct statistical comparison challenging and, in some cases, methodologically inappropriate. Furthermore, some of the datasets collected during the HOOP project are confidential and cannot be fully disclosed or subjected to open statistical treatment. Nevertheless, we recognize the value of deeper analysis. While the current manuscript focuses on presenting and validating the indicator framework and baseline results, we hope that future studies by researchers and practitioners will take advantage of the publicly available tools developed by the HOOP project to conduct more sophisticated inter-city or longitudinal analyses.
- Limitations: We fully agree that differences in data availability and quality constitute a key limitation of the study. The baseline assessment for the eight participating cities required compiling information from multiple municipal departments and local stakeholders, which naturally led to variations in the completeness, level of detail and structure of the data provided. This heterogeneity reflects the differing data management practices, governance structures and monitoring capacities of each city. Furthermore, the framework integrates both quantitative and qualitative variables related to governance, awareness and policy implementation. While this mixed-methods approach is essential to capture the multidimensional nature of urban bioeconomy, it also introduces challenges in terms of ensuring comparability, particularly for qualitative enabling factors, where interpretations can vary across local contexts. Some detailed datasets used during the project were shared by cities under confidentiality agreements and therefore cannot be disclosed or analyzed beyond aggregated levels. This confidentiality constraint also limits the extent to which the study can harmonize certain data elements or perform deeper statistical comparisons. In response to the reviewers’ recommendations, the revised manuscript now includes a dedicated paragraph in the Discussion, “Limitations in data availability and comparability”, explicitly addressing these limitations, acknowledging: the heterogeneity in data availability across cities, differences in quality and the intrinsic challenges of comparing qualitative indicators across diverse urban contexts.
- Policy relevance: We agree that strengthening the policy relevance of the study can enhance its usefulness for local authorities and practitioners. In response, the revised manuscript now includes a “Policy Implications” subsection in the Discussion section. This addition outlines how the proposed indicators can support municipal decision-making processes, particularly in the prioritization of circular bioeconomy actions and the evaluation of existing waste and resource management strategies. The new subsection highlights which indicator categories are most directly actionable for policymakers, specifically, those related to Waste, Policy and Society dimensions, and provides guidance on how these indicators can be operationalized. By translating the indicator framework into concrete areas of action, the revised manuscript aims to offer a clearer decision-support perspective for municipalities seeking to implement or enhance circular bioeconomy measures. We believe this intensifies the practical value of the study for non-academic audiences. 
- Definition of urban bioeconomy: We agree that a clearer and more operational definition of the urban bioeconomy is essential to distinguish it from the broader bioeconomy framework and to clarify the conceptual foundations of our indicator set. In response, we have expanded the Introduction to include a dedicated paragraph that defines the urban bioeconomy as the valorization of urban biowaste and wastewater through circular solutions that enable local bio-based value chains and contribute to resource efficiency, decarbonization and social well-being at city level. This definition builds on the conceptual work developed within the HOOP project and explicitly frames cities as active enablers of localized bio-based circularity. By emphasizing the transformation of urban waste streams into resources within local economic systems, the revised introduction highlights the specificities of the urban context and addresses the conceptual gap noted by the reviewers. This addition also clarifies how the definition underpins the development of the indicators framework presented in this study.
- Methodological weakness: The methodological approach applied in this study was intentionally designed to fit the objectives and constraints of the HOOP project, which required developing a practical and operational indicator framework applicable across eight highly heterogeneous European cities. For this reason, the work followed a criteria-driven and expert-informed process, rather than a data-driven methodology such as machine learning or text analytics. The indicator development relied on: a comprehensive review of existing circular economy frameworks, a pre-screening based on relevance to urban bioeconomy, an assessment of data availability and comparability across cities and expert consultation through the Local HOOP Committees. This participatory method was essential to ensure that the final indicators reflected both local priorities and operational feasibility. While we acknowledge that more algorithmic or fully systematic approaches may be appropriate in contexts with highly standardized datasets, the diversity of the cities involved, combined with the confidentiality of certain technical inputs shared by local authorities, made such methodologies unsuitable for the scope of this work. The chosen approach allowed us to obtain a set of indicators that is consistent, replicable in similar urban contexts and aligned with the practical needs of municipalities advancing their circular bioeconomy strategies. We appreciate the reviewers’ suggestion and consider it a valuable direction for future academic research, particularly once more harmonized and openly accessible datasets become available at the urban level.

# Reviewer report: Reviewer response for version 1

**DOI:** 10.21956/openreseurope.21657.r60190

**Published:** 2025-10-06

**Authors:** Sebastian Leavy

**Affiliations:** 1 National Institute of Agricultural Technology—INTA-AER Vedia, Vedia, Argentina


The article effectively develops and applies the Urban Bioeconomy Indicators (UBI) to assess circular economy progress in cities, focusing on biowaste. The rationale is well explained, addressing gaps in existing frameworks, such as those of the OECD. The methodology is partially clear, with detailed steps that include a questionnaire (not included) and criteria for 20 indicators in six dimensions. 

Replication is partially feasible using the described process and formulas in Table 2. The results illustrate application in eight anonymized cities, but raw data (e.g., for figures) are not shared, limiting full reproducibility. 

It is suggested that they be stored in a repository.

 It mentions the scenario aspect, which is undoubtedly important, but does not elaborate on feedback over time. Conclusions about innovations (e.g., enabling factors, focus on material valorization) are supported by the findings.

Access to data would be important for greater transparency.

Overall, this is a valuable contribution to the assessment of urban sustainability and the urgent need to build holistic bioeconomic indicators.


Is the rationale for developing the new method (or application) clearly explained?

Yes

Is the description of the method technically sound?

Yes

Are the conclusions about the method and its performance adequately supported by the findings presented in the article?

Yes

If any results are presented, are all the source data underlying the results available to ensure full reproducibility?

Partly

Are sufficient details provided to allow replication of the method development and its use by others?

Partly

Reviewer Expertise:

Bioeconomy

I confirm that I have read this submission and believe that I have an appropriate level of expertise to confirm that it is of an acceptable scientific standard, however I have significant reservations, as outlined above.

# Response

**Published:** 2026-01-30

**Authors:** Teresa Alvariño Pereira

We sincerely thank the reviewer for the positive and constructive evaluation of our work. We appreciate the acknowledgement of the study’s contribution to advancing the assessment of urban sustainability and the development of holistic bioeconomy indicators.  Regarding the comment on reproducibility and data availability, we acknowledge that the raw datasets used in this study cannot be fully disclosed due to confidentiality agreements with the participating cities. This limitation is explicitly related to the nature of the project, as several data inputs were shared internally by municipal authorities for baselines analysis purposes only. Nevertheless, all formulas, indicator definitions and methodological steps are fully described in the manuscript, enabling external researchers to replicate the framework using their own datasets.  Regarding the suggestion to provide more detail on scenario development and temporal feedback, we agree that this is an important aspect of circularity assessment. In the context of the HOOP project, the scope of the current work was limited to establishing a harmonized baseline across the eight cities at a single point in time. The integration of scenario evolution and dynamic monitoring will indeed be a valuable area for future research, particularly as more longitudinal data become available at the urban level.  We greatly appreciate the reviewer’s constructive feedback and positive assessment, which help reinforce the relevance and clarity of the proposed Urban Bioeconomy Indicator framework.
